# Ten Years of High-Consequence Pathogens—Research Gains, Readiness Gaps, and Future Goals

**DOI:** 10.3201/eid3004.240160

**Published:** 2024-04

**Authors:** Jennifer H. McQuiston, Joel M. Montgomery, Christina L. Hutson

**Affiliations:** Centers for Disease Control and Prevention, Atlanta, Georgia, USA

**Keywords:** Ebola virus, *Zaire ebolavirus*, hemorrhagic fever, mpox, monkeypox virus, sexually transmitted infections, henipaviruses, Nipah virus, Hendra virus, viruses, zoonoses

In 2014, high-consequence pathogens were described as those causing high mortality but having infrequent spillover from animals and rare human-to-human transmission (*1*). Since then, high-consequence pathogens such as ebolaviruses, monkeypox virus (MPXV), and henipaviruses have challenged that description. Unbeknownst to the authors of the 2014 article (*1*), *Orthoebolavirus zairense* (Zaire Ebola virus) was circulating at that time in Guinea, resulting in the world’s largest known Ebola virus disease (EVD) outbreak. The West Africa EVD outbreak redefined our understanding of high-consequence pathogens, demonstrating their substantial potential for communicable spread; in Guinea, Liberia, and Sierra Leone, >28,600 persons were infected and >11,300 patients died. EVD cases were exported to 7 other countries by infected travelers and healthcare workers; containment took 2 years, a coordinated multinational effort, thousands of volunteers, and billions of dollars (*2*). The outbreak also drove medical progress; clinical trials tested new Ebola virus vaccines (*3*), and epidemiologic investigations showed filoviruses can persist in semen and spread through sexual contact (*4*).

During 2014–2024, eleven additional Ebola virus outbreaks have been recognized; the second largest known outbreak infected 3,470 persons in North Kivu, Democratic Republic of the Congo (DRC), during 2018–2020 (*5*). Virus sequencing data suggested that >3 outbreaks were most likely caused by a persistently infected survivor from a previous outbreak (*5*). Other viral hemorrhagic fevers (VHFs) also occurred. In 2019, Chapare virus caused illness in 9 patients in Bolivia, 4 of whom died; 3 healthcare workers were infected through probable nosocomial exposure, and Chapare virus was shown to persist for months in semen after acute infection (*6*). In 2022, Marburg virus was reported in Equatorial Guinea and Tanzania, infecting 24 laboratory-confirmed patients and killing 17 persons (*7*); *Orthoebolavirus sudanense* (Sudan virus) infected 164 and killed 55 persons in Uganda (*5*).

Despite achievements in Ebola virus vaccine development, similar success has lagged for medical countermeasures for other VHFs. Without vaccines or proven therapeutics, primary mitigation measures are rapid identification and isolation of case-patients, contact tracing, and strong infection control practices. Even with accessible vaccines, the response speed is key; delayed detection and response can result in larger outbreaks requiring more resources and time to control. During the 2018 DRC outbreak, regional insecurities impeded early detection and rapid response and likely contributed to the outbreak scale and complexity (*8*).

Despite successful variola virus eradication, orthopoxviruses remain high-consequence pathogens that create complex control challenges. During 2014–2024, reports of mpox caused by MPXV surged in regions where the virus is enzootic. During 2017–2022, increased reports of mpox in Nigeria initially raised little concern, even though mpox was diagnosed in occasional travelers from Nigeria who had no history of animal contact (*9*). In May 2022, MPXV clade II began circulating person-to-person globally, primarily through sexual contact among gay, bisexual, and other men who have sex with men (*10*). Thanks to smallpox preparedness work performed by the US Centers for Disease Control and Prevention and other partners, regulatory agency-approved diagnostics, the JYNNEOS vaccine (https://www.jynneos.com), and TPOXX therapeutic agent (SIGA Technologies, https://www.siga.com) were available in some countries; however, limited early supplies of the JYNNEOS vaccine and lack of licensure in some countries meant the vaccine was not accessible to all persons at risk for MPXV exposure. Before the global mpox outbreak, no real-world efficacy data for TPOXX was available; clinical trials are ongoing. After the mpox outbreak peak in 2022, MPXV has continued to circulate at low levels through 2024; since 2022, >90,000 cases have been reported worldwide (*11*). Since 2023, similar surveillance signals have been seen in DRC with MPXV clade I (*12*), raising concerns for another global mpox outbreak caused by a more lethal virus clade.

Henipaviruses are high-consequence pathogens within the Paramyxoviridae family; >600 human infections caused by Nipah and Hendra virus have been reported in the published literature (*13*). During 2014–2024, seasonal outbreaks of Nipah virus have occurred regularly in Bangladesh and India, but the geographic range of the natural reservoir hosts, *Pteropus* spp. bats, includes Bhutan, Cambodia China, Indonesia, Madagascar, Malaysia, Maldives, Myanmar, Nepal, Pakistan, Philippines, Sri Lanka and Thailand (*14*). In Bangladesh and India, infection risk factors include date palm sap production or consumption (*15*), but person-to-person transmission has also been documented among families and close contacts; nosocomial transmission to healthcare workers remains a substantial risk factor (*13*). During a 2018 outbreak in India, Nipah virus infected 23 persons with a mortality rate >90%; of those cases, 19 were attributed to person-to-person spread among healthcare workers and patients in hospital settings (*16*). Nipah virus is considered to have substantial pandemic potential because it infects multiple mammal species, is endemic in densely human populated regions, and is an RNA virus that can have high mutation rates.

Despite a decade of ecologic studies, no definitive reservoir has been identified for Ebola virus or MPXV, making it challenging to provide clear messaging to prevent animal spillover events. Investments in early detection and response in disease-endemic countries can prevent virus spread after a spillover event. In Uganda, VHF detection rates dropped from an average of 2 weeks to only 2 days after targeted investments in diagnostic capacity were initiated (*17*). Furthermore, earlier detection translated into more rapid control measure use; the average numbers of cases per outbreak dropped from >100 before to 5 cases after those investments (*17*). However, investments are difficult to sustain and require continuous training, funding, and political will.

Since the description in 2014 (*1*), the past decade has redefined high-consequence pathogens as serious and deadly agents that pose a substantial threat to domestic and global security. Many of those pathogens are contagious, most spread from animals to humans, and some can be used as bioterrorism agents. The effects of a changing climate and increasing human–animal interactions associated with population growth and agriculture, global travel and trade, political instability, and human migration events ensure outbreaks of high-consequence pathogens will continue to pose public health threats. The 10 years since 2014 have shown how person-to-person transmission of high-consequence pathogens can fuel large, complex outbreaks, emphasizing the need for swift, effective detection and control at the earliest stages of emergence.

Recognizing the value of early detection and rapid response, a 7-1-7 framework has been proposed as a global metric for pandemic prevention (*18*): 7 days to detect emergence, 1 day to notify/mobilize a response, and 7 days to implement control measures. The framework is being piloted worldwide and has been adopted as a regional strategy in Africa. However, to meet this goal, improvements in local capacity, including specimen transport, laboratory diagnostics, trained healthcare and laboratory workers, defined reporting structures, and a robust public health workforce, are urgently needed worldwide where high-consequence pathogens continue to emerge. Investments in high-containment facilities, world-class subject matter experts, and cutting edge technologies are also critical to ensure robust public health testing capabilities at home and abroad and a more rapid resolution to outbreaks caused by high-consequence pathogens.
